# Carnitine analysis in pterygium

**DOI:** 10.5935/0004-2749.20200001

**Published:** 2020

**Authors:** Ayhan Saglik, Ismail Koyuncu, Hamza Yalcin, Fatih Mehmet Adibelli, Ataman Gonel, Muslum Toptan

**Affiliations:** 1 Department of Ophthalmology, Harran University Faculty of Medicine, Şanlıurfa, Turkey; 2 Department of Biochemistry, Harran University Faculty of Medicine, Şanlıurfa, Turkey; 3 Unit of Biometry and Genetics, Harran University Faculty of Agriculture, Şanlıurfa, Turkey

**Keywords:** Acylcarnitine, Gas chromatography-mass spectrometry, Carnitine, metabolomics, Pterygium, Acetilcarnitina, Cromatografia gasosa-espectrometria de massas, Carnitina, Metabolômica, Pterígio

## Abstract

**Purpose:**

The aim of the present study was to measure the free carnitine and acylcarnitine levels in pterygium tissue and normal conjunctival tissue at the metabolomics level using tandem mass spectrometry.

**Methods:**

In this prospective, clinical randomized study, pterygium tissues and normal conjunctival tissues taken during pterygium excision with autograft were compared regarding their free carnitine and acylcarnitine profiles. After tissue homogenization, carnitine levels were measured using tandem mass spectrometry. The data were statistically analyzed with the Wilcoxon signed-rank test.

**Results:**

Pterygium and normal conjunctival tissue samples from a single eye of 29 patients (16 females, 13 males; mean age, 54.75 ± 11.25 years [range, 21-78 years]) were evaluated. While the free carnitine (C0) level was significantly high in the pterygium tissue (p<0.001), acylcarnitine levels were significantly high in some esterized derivatives (C2, C5, C5:1, C5DC, C16:1, C18, methylglutarylcarnitine) (p<0.05). No statistically significant difference was determined for the other esterized derivatives (p>0.05).

**Conclusion:**

That the carnitine levels in pterygium tissue were higher suggests that acceleration of cell metabolism developed secondary to chronic inflammation and the premalignant characteristics of pterygium tissue. High carnitine levels may also effectively suppress the apoptosis process. The data reported in our study indicate that further, more extensive studies of the carnitine profile could help clarify the pathogenesis of pterygium.

## Abstract (pt)

**Objetivo:**

O objetivo deste estudo foi medir os níveis de carnitina livre e acil-carnitina a nível metabolómico com espectrometria de massa em tandem no tecido do pterígio e no tecido conjuntivo normal.

**Método:**

Neste estudo prospetivo, clínico e aleatório, os tecidos de pterígio e os tecidos normais de conjuntiva, retirados durante a cirurgia de pterígio com autoenxerto, foram comparados em relação ao perfil de carnitina livre e de acil-carnitina. Após a homogeneização dos tecidos, os níveis de carnitina foram medidos por espectrometria de massa em tandem. A análise estatística dos dados foi realizada com o teste dos postos sinalizados de Wilcoxon.

**Resultados:**

A avaliação foi feita através de amostras de tecido pterígio e de conjuntiva normal de um único olho de 29 pacientes (16 mulheres, 13 homens). A média de idade dos pacientes foi de 54,75 ± 11,25 anos (faixa dos 21 aos 78 anos). Enquanto o nível de carnitina livre (C0) foi significativamente elevado no tecido pterígio (p<0,001), os níveis de acil-carnitina foram significativamente elevados em alguns derivados esterificados (C2, C5, C5: 1, C5DC, C16:1, C18, metilglutaril carnitina) (p<0,05). Não foi determinada uma diferença estatisticamen te significante noutros derivados esterificados (p>0,05).

**Conclusão:**

Os níveis mais elevados de carnitina no tecido do pterígio sugerem que a aceleração do metabolismo celular se tenha tornado secundária com o efeito da inflamação crónica e o caráter pré-maligno do tecido do pterígio. Os níveis elevados de carnitina também podem ser eficazes na supressão do processo de apoptose. Os dados obtidos no estudo indicam que estudos mais extensivos do perfil da carnitina contribuiriam para o esclarecimento da patogénese do pterígio.

## INTRODUCTION

Pterygium is defined as a benign fibrovascular mass that begins at the bulbar conjunctiva and extends in the form of a wing to the cornea. Pterygium tissue that extends to the cornea can lead to corneal surface impairments and vision loss and result in poor cosmesis^(1^,^2)^.

Hereditary factors and several environmental factors, such as ultraviolet (UV) damage and human papillomavirus (HPV) infection, are reported to play a role in the pathogenesis of pterygium. Chronic inflammation and DNA damage in pterygium tissue result in uncontrolled cell proliferation, tissue invasion, and local angiogenesis^(1^,^3^-^5)^. High recurrence rates of p53 tumor suppressor gene and KRAS oncogene mutations have also been supported in the etiology of pterygium^(6^,^7)^. Uncontrolled proliferation associated with inhibition of the apoptosis pathway in pterygium tissue can alter the metabolic balance in the cells^(8^,^9)^.

Recently, more attention has been given to metabolomics studies with the aim of obtaining new biochemical markers in metabolism. Using highly advanced technologies, metabolomics identifies and determines the amount of metabolites in the cell cycle^(10)^. This method is widely used in assessing carnitine metabolism.

Carnitine (β-hydroxy-γ-trimethylammonium butyrate) is a branched, non-essential amino acid, synthesized from essential amino acids such as lysine and methionine. Typically, 75% of the daily carnitine requirement is provided by the meat and dairy products comprising the adult dietary requirement, and the remainder is synthesized endogenously^(11)^. The presence of free carnitine (FC) and β-oxidation of fatty acids is essential for the production of energy and acylcarnitines (AC)^(12)^. By binding to long-chain fatty acids of acyl residues, FC is involved in mitochondrial transfer for β-oxidation. Organic acidemia and impairments in fatty acid metabolism develop as a result of carnitine deficiency. Moreover, deficiencies may also result in fatty acid accumulation in organelles such as peroxisomes, microsomes, and mitochondria^(13)^.

In this metabolomics study, the FC and AC profiles were evaluated in pterygium tissue and normal conjunctival tissue using liquid chromatography coupled to mass spectrometry. (Shimadzu Corporation, Japan).

Examination of the carnitine profile, which is frequently performed in investigations of inflammatory and metabolic disease, is considered an important milestone in the pathophysiology and treatment of pterygium.

## METHODS

This prospective study evaluated tissue samples from a single eye of 29 patients with pterygium (16 females and 13 males). The study was approved by the Ethics Committee of Harran University Faculty of Medicine, Şanlıurfa. All procedures were performed in accordance with the principles of the Declaration of Helsinki. Informed consent was obtained from all of the participants.

We excluded patients who had used systemic immunosuppressant drugs or topical treatment (steroid and cyclosporine eye drops) for the eyes in the 2 weeks before the procedure, if they had a history of ocular surgery, or if they had ocular malignancy, uveitis, conjuntivitis, glaucoma, or systemic inflammatory disease.

Visual acuity measurements and biomicroscopic examinations were performed, and anterior segment photographs were taken before and after the procedure. The pterygium was classified clinically in three types, as described by Ozturk et al.^(7)^. In Type 1, the pterygium tissue had not passed the limbus; in Type 2, the pterygium tissue was between the limbus and the optic zone; and in Type 3, the pterygium tissue had reached the optic zone. In patients with bilateral pterygium, the eye with the more advanced pterygium was evaluated.

The surgical procedure was performed using the standard conjunctival autograft transplantation technique with 8.0 vicryl sutures^(14)^. We took 1 × 1 mm tissue samples from the pterygium and superotemporal bulbar conjunctival region from each patient. The samples were separately placed in Eppendorf tubes and stored at -80°C until measurement of the carnitine levels.

### Laboratory procedures

We evaluated the carnitine profile by modifying the neonatal screening method developed by la Marca et al.^(15)^ and Azzari et al.^(16)^. Equal amounts of each tissue were excised from each patient, and analyses were performed. The pterygium and normal conjunctival tissue samples were washed with cold PBS (buffer solution) (Sigma Aldrich, USA). After adding RIPA lysis buffer (10 mM Tris-HC1 pH = 8, 1 mM EDTA, 1 mM EGTA, 140 mM NaCl, 1% Triton X-100, 0.1% SDS, 0.1% sodium deoxycholate) to the tissues, the samples were homogenized for 1 h at 4°C (Qiagen TissueLyser). The supernatant was transferred into new tubes by centrifuging the tissue lysate at 4°C for 10 min at 12,000 *g*.

Filter papers cut into 3.2 mm disks (Whatman filter paper 10538018) were placed in 96-well plates. A 5 µL tissue homogenate sample was added and left to dry overnight at room temperature. The spot sample was extracted and butylated by dispensing 300 µL of an extraction solution comprised of a methanol and aqueous solution of 3 mmol/L hydrate hydrazine at 37°C for 25 min.

For internal standards, stable heavy isotope analogs of carnitine and acylcarnitine (Labeled Carnitine Standards Set B [Cambridge Isotope Laboratories]) were used in the extracted solution. The extracted samples were injected into the LCMS-8040 (Shimadzu Corporation, Japan) device. The percentage of each analyte was defined by comparison with an internal standard. Standard concentrations of acylcarnitine ranged from 7.6 to 152 µmol/L with a run of 2.2 min in FIA Flow 0.070 µL/min (A: water + 0.05% formic acid; B: acetonitrile, A/B: 30%/70%). We injected 40 µL of the sample into the column oven at 30°C; the desolvation line was 300°C, heat was 500°C, nebulizing gas was 3 L/min, and drying gas was 20 L/min.

The residual pellet was lysed in 0.25 µL lysis buffer, the protein concentration of which was identified with the BCA protein assay kit (Thermo Fisher Scientific, Waltham, MA, USA). Finally, total protein levels were normalized and net carnitine levels in the supernatants defined. All of the data collected were reprocessed using Shimadzu Neonatal Software, which automatically calculates the concentration of each component.

### Statistical analysis

All data analyses were conducted using SPSS Statistics for Windows, Version 24 (SPSS Inc., NY, USA). Conformity of the data to normal distribution was tested using the Shapiro-Wilk test. Because no data showed normal distribution, we used the median and interquartile range (IQR). The Wilcoxon signed-rank test was used to determine the differences between the two independent groups. For all tests, a p value of <0.05 was considered statistically significant.

## RESULTS

The mean age of the patients was 54.75 ± 11.25 years (range, 21-78 years). Type 2 pterygium was identified in 24 (83%) eyes and Type 3 pterygium in 5 (17%) eyes. The pterygium was located in the nasal region in all cases. The study was conducted in a geographical region with a hot climate and high exposure to UV light. Analyses were performed on the pterygium and normal conjunctival tissues that were taken from the patients during pterygium surgery with conjunctival autograft transplantation.

Before treatment, cataract was identified in five patients and degenerative myopia in one. The corrected distance visual acuity level was statistically significantly increased 6 months after treatment (p<0.01). No patient developed postoperative complications or recurrence.

**Figure 1:**
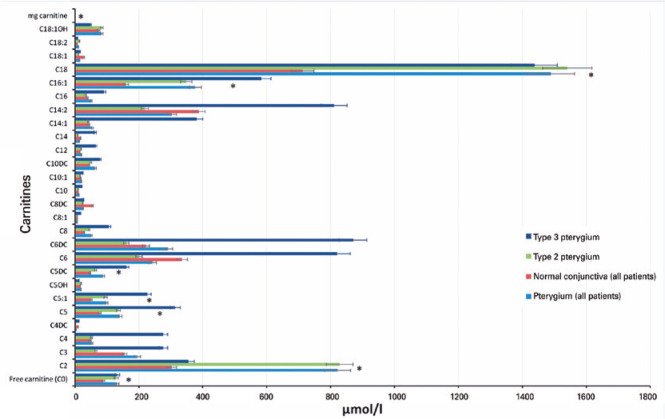
Median tissue concentrations of carnitines in all pterygium tissues, normal conjunctival tissues, and Type 2 and 3 pterygium tissues (with s.e. bars).

The FC (C0) level was significantly high in the pterygium tissue (p<0.001) as well as some AC in the form of esterized derivatives. These were C2, C5, C5:1, C5DC, C16:1, C18, and methylglutarylcarnitine (p<0.05) (Figure 1, Table 1). Regarding the AC esters C3, C4, C5OH, C6DC, C8, C10, C10:1, C10DC, C12, C14:1, C16, C18:2, and C18:1OH, although the levels were higher in the pterygium tissue than in the normal conjunctival tissue, the difference was not statistically significant (Table 1). The levels of C4DC, C6, C8:1, C8DC, C14, C14:2, and C18:1 were higher in the normal conjunctival tissue, but the difference was not statistically significant (p>0.05) (Table 1).

**Table 1:** Mean and median levels of carnitines in the pterygium and control groups

n: 29	Median (µmol/l)	IQR	Mean (µmol/l)	SD ±	P Values
C0 ^*^ Pt	131.14	273.52	223.86	202.80	<0.001
Nc	88.39	113.44	124.30	101.41	
C2 ^*^ Pt	821.74	1486.79	1362.26	1547.96	<0.001
Nc	302.52	294.86	596.54	812.60	
**C3** Pt	195.72	518.24	278.86	317.02	= 0.107
Nc	154.86	723.01	361.96	438.43	
**C4** Pt	52.39	87.89	76.53	87.34	= 0.174
Nc	47.33	88.83	68.27	73.42	
**C4DC** Pt	6.05	18.99	15.47	20.14	= 0.443
Nc	9.80	27.00	18.96	22.50	
C5 ^*^ Pt	140.36	256.57	267.65	376.62	=0.001
Nc	77.16	104.98	130.18	184.29	
C5:1 ^*^ Pt	98.15	114.86	126.64	106.54	<0.001
Nc	50.51	64.91	70.90	78.53	
**C5OH** Pt	17.99	49.55	41.09	55.33	=0.055
Nc	16.03	39.22	31.19	39.13	
C5DC ^*^ Pt	87.39	186.78	138.97	176.63	<0.001
Nc	46.98	114.02	64.49	65.22	
**C6** Pt	242.84	1262.64	1156.87	2588.98	=0.123
Nc	335.03	756.35	670.13	1417.84	
**C6DC** Pt	291.16	896.03	719.45	1347.17	=0.088
Nc	222.44	747.36	436.70	516.88	
**C8** Pt	50.32	187.72	119.75	179.26	=0.135
Nc	28.22	125.29	97.34	155.60	
**C8:1** Pt	6.59	17.03	15.34	20.44	=0.737
Nc	6.88	10.25	15.80	33.21	
**C8DC** Pt	25.11	168.39	133.19	197.88	=0.693
Nc	55.29	113.16	97.55	122.37	
**C10** Pt	11.58	24.64	20.54	21.15	=0.143
Nc	10.17	12.13	12.97	13.62	
**C10:1** Pt	20.54	49.61	47.68	71.05	=0.304
Nc	17.68	40.46	46.09	82.35	
**C10DC** Pt	63.41	90.02	86.35	92.41	=0.424
Nc	43.52	100.22	92.89	121.21	
**C12** Pt	20.26	49.18	54.15	94.98	=0.115
Nc	15.11	42.23	40.43	69.05	
**C14** Pt	13.56	62.12	61.78	111.90	=0.274
Nc	16.91	105.91	60.51	85.53	
**C14:1** Pt	54.83	98.76	134.21	216.91	=0.191
Nc	44.30	94.55	81.06	93.70	
**C14:2** Pt	302.70	918.42	602.02	599.82	=0.107
Nc	388.22	982.18	752.19	719.57	
**C16** Pt	51.20	137.83	101.35	132.30	=0.889
Nc	39.90	161.56	106.23	106.23	
C16:1 ^*^ Pt	376.57	644.29	614.19	784.27	<0.001
Nc	159.33	387.67	240.06	235.79	
C18 ^*^ Pt	1489.41	3280.73	2192.08	2029.48	<0.001
Nc	712.35	1102.47	1039.21	979.75	
**C18:1** Pt	14.21	113.58	64.17	92.99	=0.638
Nc	27.90	96.45	58.16	69.59	
**C18:2** Pt	10.77	54.66	39.15	60.45	=0.316
Nc	7.05	61.46	40.25	61.51	
**C18:1OH** Pt	83.18	166.47	128.53	148.28	=0.886
Nc	74.59	147.50	122.68	151.60	
Mg ^*^ Pt	2.22	4.94	3.68	3.83	<0.001
Nc	1.23	2.29	1.67	1.92	

C0= free carnitine; mg= methylglutarylcarnitine; Nc= normal conjunctiva; IQR= interquartile range; Pt= pterygium; p value= obtained by Wilcoxon signed-rank test; SD= standard deviation.

* p<0.05.

*= p<0.05.

mg carnitine= methyl glutarylcarnitine.

The distributions of FC and AC levels of all patients are shown as a bar plot in figure 1. The majority of AC elevation was in long-chain (18 carbon length). In Type 3 pterygium, an increase was determined to be in range (3-16 carbon length), and elevated C2, C18, and C18:1OH values were noted in Type 2 pterygium.

**Figure 2:**
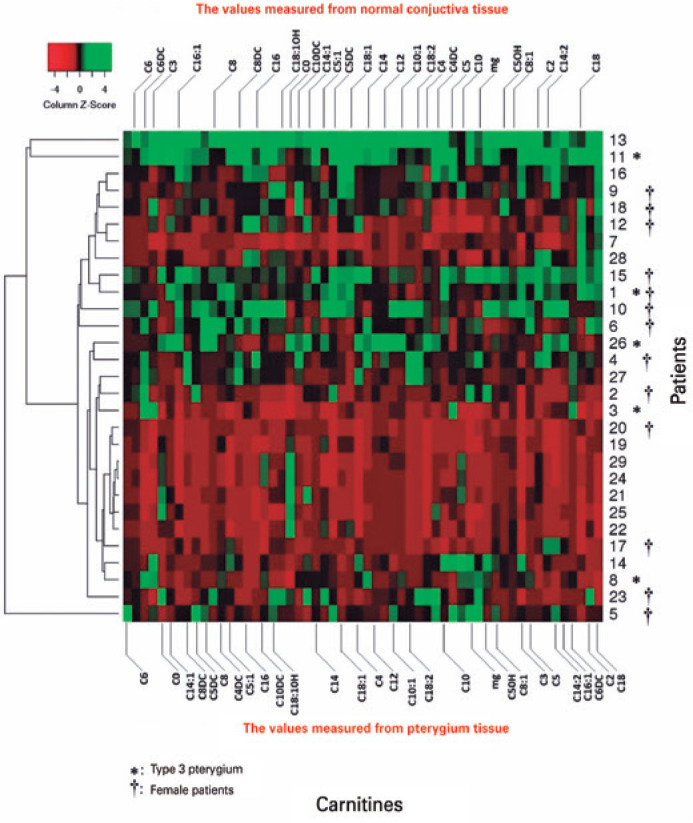
Median carnitine concentrations on heat map analysis. The upper part of the map contains data on normal conjunctival tissues, and the lower part of the map contains data on pterygium tissues.

The distribution of carnitine values is shown as a heatmap in figure 2. The red coloring on the map shows that the low values are clustered in the center section. These data are formed from the common characteristics of Type 2 pterygium and male patients. In the upper section of the map, the green portion displays the high carnitine values of patients 11 and 13. One of these patients was Type 2, and the other was Type 3.

* Type 3 pterygium patients.

mg carnitine= methylglutaryl carnitine; C0= free carnitine.

## DISCUSSION

Differences exist in the metabolic needs of pterygium cells compared with normal conjunctiva due to the fibrovascular proliferation process, UV damage, and chronic inflammation. These processes may also affect carnitine metabolism, which shows abnormality in several inflammatory and metabolic diseases. Therefore, in the current study, we performed a metabolomics analysis of the FC and AC levels, which are basic components of the transport of cellular long-chain fatty acids to the mitochondria^(17)^.

Fibrovascular proliferation, which develops through matrix metalloproteinases (MMP), is shown to be associated with inflammation in pterygium tissue and plays a role in shaping pterygium as a result of triggering inflammatory processes^(1^,^3^,^18)^. In addition, inflammation negatively affects carnitine metabolism in the cells. Previous studies have reported that plasma carnitine levels are reduced in inflammatory processes in particular^(19^,^20)^. In the current study, carnitine levels were analyzed in tissue extracts, and FC levels were significantly increased, particularly at the tissue level (Table 1). The increase in carnitine levels is considered to relate to the acceleration of some metabolic processes and the increased need for energy in the cell in the process of chronic inflammation in the pterygium tissue.

In a dry eye study, a disease in which inflammation is considered to be a factor, L-carnitine inhibited mitogen-activated protein kinase activation, which otherwise stimulates inflammatory cytokines, chemokines, and MMPs. This was reported to be effective in protecting against the destructive effects of UV damage (redness, pain, melanin production, collagen damage)^(21)^. Hua et al.^(22)^ reported that L-carnitine protected human corneal epithelial cells by its antioxidant effect and by suppressing ROS production, thus preventing membrane lipid oxidation and mitochondrial DNA damage.

In the present study, lower levels of C4DC, C6, C8:1, C8DC, C14, C14:2, and C18:1 were observed in pterygium tissue compared with normal conjunctival tissue, whereas other AC esters were higher (Figure 1). Notably, statistically significantly higher levels of the ester carnitines C2, C5, C5:1, C5DC, C16:1, C18, and methyl glutaryl were found in the pterygium tissue. This significant difference could be associated with the metabolic activity of new signal foci causing pterygium tissue proliferation and carnitines, which indirectly function as transport in energy provision with the transfer of fatty acids to the mitochondria. While 99% of carnitines are within the cell, levels of FC and AC in the blood circulation are demonstrated in the homeostasis of the whole organism^(12)^. Although several factors exist at the molecular level in the pathogenesis of pterygium, that FC and AC levels were found to be high compared with the normal conjunctiva suggests that the process of carnitine metabolism is accelerated at the molecular level.

A study examining peroxisome mediation of antioxidant levels in conjunctival and pterygium tissues demonstrated that mitochondrial DNA was most affected by UV damage^(23)^. Impaired mitochondrial function plays a critical role in pathological conditions such as hypoxia-ischemia damage, stroke, and diabetes.

Examination of the heatmap data reveals clustering of the carnitine values, particularly in Type 2 pterygium and male patients, more in the central area. However, due to the small number of patients in the Type 3 pterygium group, it was not possible to evaluate the dis tribution (Figure 2).

In conclusion, uncontrolled cell proliferation, normal tissue invasion, tumor suppressor gene, and oncogene mutations are known to occur in pterygium pathogenesis. The present study demonstrates higher carnitine levels in pterygium tissue than in normal conjunctival tissue, suggesting that the effect of premalignant cells and chronic inflammation lead to an acceleration in metabolism. In addition, high carnitine levels may be effective in suppressing the apoptosis process in pterygium tissues.

Overall, the carnitine profile could shed new light in understanding pterygium pathophysiology. Further studies of the carnitine profile in the pterygium are necessary using a larger patient series in order to halt proliferation and reduce recurrence rates after surgery.
